# MULTIMERIN2 binds VEGF-A primarily via the carbohydrate chains exerting an angiostatic function and impairing tumor growth

**DOI:** 10.18632/oncotarget.6515

**Published:** 2015-12-09

**Authors:** Roberta Colladel, Rosanna Pellicani, Eva Andreuzzi, Alice Paulitti, Giulia Tarticchio, Federico Todaro, Alfonso Colombatti, Maurizio Mongiat

**Affiliations:** ^1^ Department of Translational Research, Experimental Oncology Division 2, CRO, Aviano, Italy

**Keywords:** extracellular matrix (ECM), angiogenesis, tumor microenvironment, vascular endothelial growth factor (VEGF), endothelial cell sprouting

## Abstract

Angiogenesis is a key process occurring under both physiological and pathological conditions and is a hallmark of cancer. We have recently demonstrated that the extracellular matrix (ECM) molecule MULTIMERIN2 exerts an angiostatic function through the binding to VEGF-A. In this study we identify the region of the molecule responsible for the binding and demonstrate that the interaction involves the carbohydrate chains. MULTIMERIN2 interacts with other VEGF-A isoforms and VEGF family members such as VEGF-B, -C, -D and PlGF-1 suggesting that the molecule may function as a reservoir for different cytokines. In response to VEGF-A_165_, we show that MULTIMERIN2 impairs the phosphorylation of VEGFR2 at both Y1175 and Y1214 residues, halts SAPK2/p38 activation and negatively affects endothelial cell motility. In addition, MULTIMERIN2 and its active deletion mutant decrease the availability of the VEGFR2 receptor at the EC plasma membrane. The ectopic expression of MULTIMERIN2 or its active deletion mutant led to a striking reduction of tumor-associated angiogenesis and tumor growth. In conclusion, these data pinpoint MULTIMERIN2 as a key angiostatic molecule and disclose the possibility to develop new prognostic tools and improve the management of cancer patients.

## INTRODUCTION

Angiogenesis, the formation of new blood vessels from preexisting vessels, is a key process and is required during reproduction, development, and wound repair [1,2]. The development of new vessels also plays a critical role in the onset and progress of many diseases, including cancer [3]. Pathological angiogenesis is a hallmark of neovascular diseases [4] and many anti-angiogenic strategies have been proposed to impair cancer growth [5]. However, these approaches were not as effective as hoped for [6–13]. To improve the therapeutic outcome, an emerging alternative is to readdress the aberrant tortuous and leaky vessels associated with tumors towards a normalized more efficient vasculature [14–19]. Angiogenesis is a tightly regulated process and involves the interaction among different cell types, several cytokines [20] and growth factors, and extracellular matrix (ECM) constituents. Fibroblast growth factors (FGFs) and vascular endothelial growth factors (VEGFs) have a recognized role in promoting angiogenesis and anti-angiogenic strategies have been developed based on these molecules [21,22]. Nonetheless, the role of ECM in affecting the development of blood vessel has also been acknowledged [23–28]. The list of ECM molecules affecting angiogenesis is large and includes different collagens [29,30], fibronectin [31,32], vitronectin [33], laminins [34], thrombospondin [35], SPARC [36], perlecan [37] and decorin [38]. To further complicate this scenario, ECM proteolytic fragments also can affect angiogenesis, often exerting opposite effects compared to the intact molecule of origin [25,39–43].

MULTIMERIN2 (MMRN2) is a pan-endothelial ECM member of the EDEN (**E**MI **D**omain **EN**dowed) protein family [44] associated with a high molecular weight glycoprotein complex [45]. The molecule is deposited along the endothelium, both in normal and tumoral vasculature, including hot spots of neovascularization in some tumors [45–48]. MMRN2 is found in tight juxtaposition with ECs, being present also in the luminal side of the vessels [45], hence suggesting an important function in EC homeostasis. Accordingly, MMRN2 exerts an angiostatic effect through a direct binding to VEGF-A [49]. In this study we identify the region of the molecule responsible for the binding to VEGF-A and demonstrate that the interaction partially occurs through the carbohydrate chains. We also found that MMRN2 interacts with other VEGF-A isoforms and VEGF family members. The effects of the active fragment on ECs biology and tumor growth closely resemble those of the entire molecule.

## RESULTS

### Identification of the region of MMRN2 responsible for the angiostatic effects

**Figure 1:**
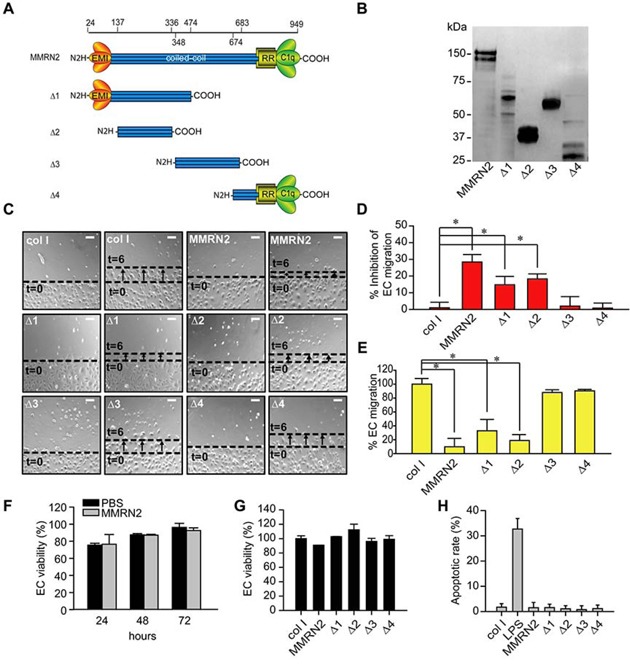
The MMRN2 functional angiostatic portion resides in the coiled-coil region **A.** Schematic representation of the various deletion mutants (Δ1 to Δ4) created and expressed in 293-EBNA cells. The numbers of the amino acid residues of the deletions is reported on top and excludes the first 24 residues of the signal peptide. The EMI domain (EMI), the coiled-coil region, the arginine-rich domain (RR) and the gC1q domain (C1q) are indicated. **B.** Western blot analysis of the His-tagged MMRN2 molecule and the various recombinant deletion mutants (Δ1 to Δ4) purified by means of the Ni-NTA resin. An anti-His antibody was used for the analysis. **C.** Representative images of the scratch test performed on HUVEC cells challenged with equimolar concentrations (35 nM) of MMRN2 and the various recombinant deletion mutants (Δ1 to Δ4); type I collagen was used as control and the front of cells at time zero (t=0) migration after 6 hours of migration (t=6) are highlighted; scale bar = 145 μm. **D.** Graph representing the analysis of the scratch test expressed as the % of inhibition of EC’ migration respect to the collagen control; (*P ≤ 0.026). **E.** Graph representing the migration on transwells of HUVEC cells challenged with equimolar concentrations (35 nM) of type I collagen (col I), MMRN2 or the deletion mutants (Δ1 to Δ4); (*P ≤ 0.001). **F.** Graph representing the % of cell viability of HUVEC cells challenged with 35 nM of MMRN2 or vehicle (PBS), following 24, 48 and 72 hours of incubation, as obtained by MTT assays. **G.** Graph representing the % of cell viability of HUVEC cells challenged with equimolar concentrations (35 nM) of type I collagen (col I), MMRN2 or the deletion mutants (Δ1 to Δ4) as obtained by MTT assays performed after 48 hours of incubation. **H.** Graph representing the % of apoptotic HUVEC cells challenged with equimolar concentrations of type I collagen (col I), MMRN2 or the deletion mutants (Δ1 to Δ4) as obtained by TUNEL assays performed after 48 hours of incubation; 100 ng/ml of LPS were used to induce apoptosis. P values were obtained with the ANOVA one way analysis of variance and graphs represent the mean ± SD obtained from at least three experiments.

In order to pinpoint the region of MMRN2 responsible for the anti-angiogenic effects, we generated a series of deletion mutants and expressed them in 293-EBNA cells (Figure 1A). The purified His-tagged deletion mutants were analyzed by Western blot (Figure 1B) and employed at equimolar concentrations (35 nM) to challenge HUVEC cells during cell migration. Similarly to the whole molecule, the Δ1 and Δ2 mutants were able to significantly inhibit the migration of HUVEC cells in scratch assays, whereas the Δ3 and the Δ4 deletion mutants did not affect cell motility (Figure 1C and 1D). This result was also corroborated by cell migration assays performed on transwells (Figure 1E). On the contrary, MMRN2 did not significantly affect the proliferation of HUVEC cells over time (Figure 1F) and these results were in accordance with the fact that it did not alter Akt phosphorylation (data not shown). Accordingly, MMRN2 and the deletion mutants did not affect the EC viability or the apoptotic rate following 48 hours of treatment (Fig. 1G and 1H).

**Figure 2:**
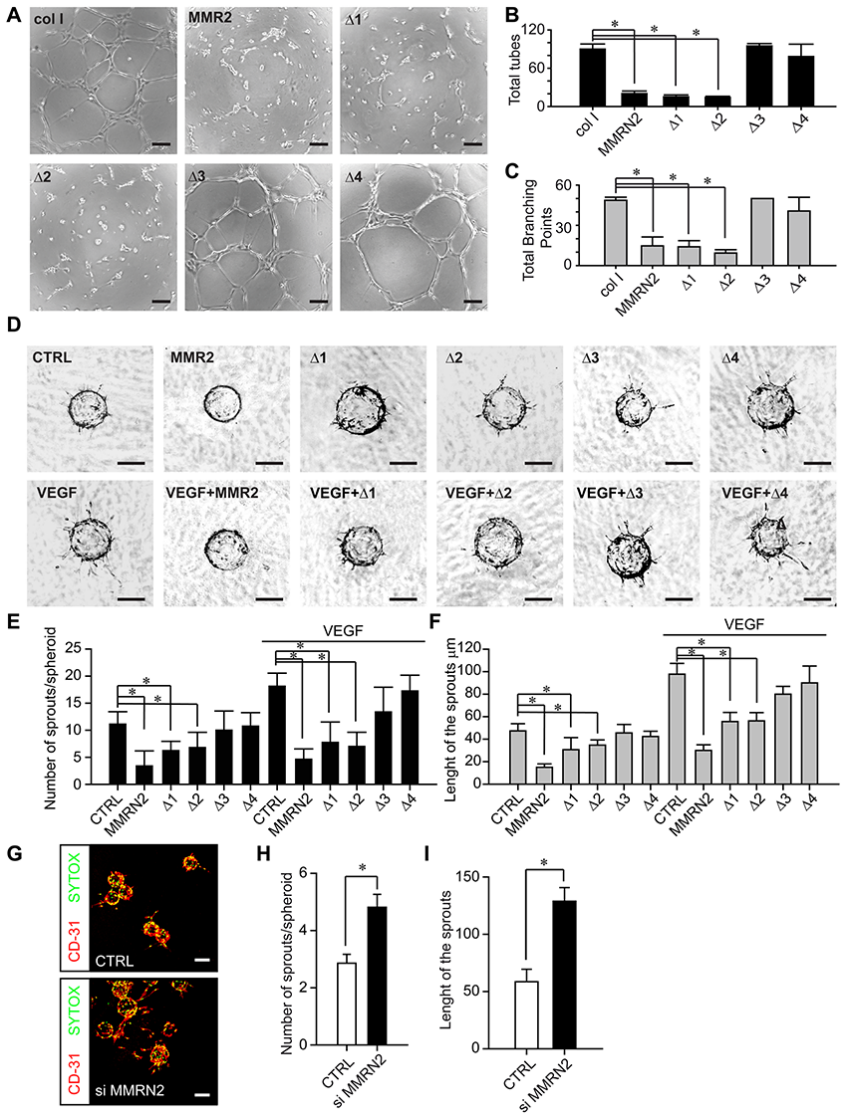
MMRN2 and the functional fragments affect EC behavior in 2D and 3D contexts **A.** Representative images of the Matrigel tubulogenesis assay upon treatment of HUVEC cells with equimolar concentrations (35 nM) of the various recombinant molecules under analysis. Type I collagen was used as control; scale bar = 100 μm. **B.** and **C.** Graphs representing the evaluation of the number of tubes and total branching points, respectively, from the experiment reported in A as obtained with the Wimasis tube analysis software; (*P < 0.001). **D.** Representative images of the spheroid angiogenesis assay obtained following coating of HUVEC cells onto cytodex microcarriers embedded into a fribrin gel overlaid with normal human dermal fibroblasts (NHDF) to induce EC sprouting. Spheroids were challenged with a 35 nM concentration of MMRN2 and the various deletion mutants in the presence or not of VEGF. Untreated spheroids served as negative control (CTRL); scale bar = 100 μm. **E.** and **F.** Graphs representing the evaluation of respectively the number and length of the sprouts of the experiment in D, as obtained with the Image J software; (*P < 0.001). **G.** Representative images of the spheroid angiogenesis assay obtained following coating of HUVEC cells transduced with the control or siMMRN2 adenoviral vectors. Fixed spheroids were stained with α-CD31 (ECs) and SYTOX (nuclei), scale bar = 160 μm. **H.** and **I.** Graphs representing respectively the number of sprouts per spheroid (*P = 0.006) and the length of the sprouts (*P = 0.009) of the experiment in (G) as assessed by the Volocity 3D software. P values from (B) (C) (E) and (F) were obtained with the ANOVA one way analysis of variance, P values from (H) and (I) were obtained were obtained using the Student's t-test and graphs represent the mean ± SD obtained from at least three experiments.

To further assess the influence of MMRN2 or its deletion mutants in affecting EC behavior, we carried out a tube formation assay on Matrigel challenging the cells with the various recombinant proteins. Either the entire molecule or the Δ1 and Δ2 deletion mutants strongly impaired the formation of tubules (Figure 2A to 2C and Figure S1A to S1D). We next applied the spheroid-based 3D angiogenesis test, better resembling the physiological condition. To this end, EC spheroids embedded in a fibrin gel and overlaid with fibroblasts were challenged with the recombinant molecules under analysis. As shown in Figure 2D, MMRN2 and the Δ1 and Δ2 mutants strongly hampered the sprouting of ECs induced by the fibroblast-derived cytokines; both the number and the length of the sprouts were decreased and the effect was even stronger in the presence of VEGF-A_165_ (Figure 2E and 2F). Accordingly, the down-modulation of MMRN2 expression increased vessels’ sprouting in 3D (Figure 2G to 2I).

### The glycosylation of MMRN2 is required for optimal interaction with VEGF-A

**Figure 3:**
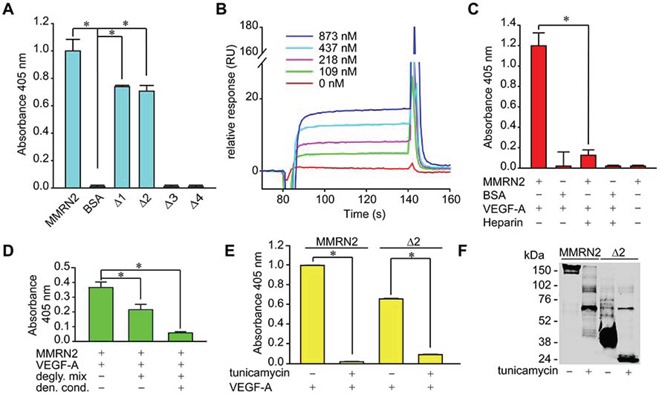
The binding of VEGF-A to MMRN2 occurs through the carbohydrate chains of the coiled-coil region **A.** Graph representing the solid phase analysis of VEGF-A_165_ interaction with the various deletion mutants indicating that the binding occurs within the coiled-coil region encompassed by the Δ2 fragment. BSA was used as negative control; (*P < 0.001). **B.** Sensogram expressed in resonance units (RU) of the surface plasmon resonance analysis of the interaction of VEGF-A_165_ (the different concentrations used are indicated) with the Δ2 deletion mutant; kD = 4.3 × 10^−7^ M. **C.** Graph representing the solid phase analysis of the interaction of VEGF-A_165_ with MMRN2 in the presence of heparin that completely abolished the interaction. BSA was used as negative control; (*P < 0.001). **D.** Graph representing the solid phase analysis of the MMRN2/VEGF-A_165_ interaction following the cleavage of the carbohydrate chains with the Protein Deglycosylation Mix (degly. mix) under non denaturing or denaturing conditions (den. cond.); (*P < 0.003). **E.** Graph representing the solid phase analysis of the interaction of MMRN2 and the Δ2 deletion mutant with VEGF-A_165_ with the employment of the recombinant molecules produced in the absence or in the presence of tunicamycin to prevent their glycosilation. The absence of the carbohydrate chains completely abolishes the interaction; (*P < 0.001). **F.** Image of the Western blot analysis of recombinant MMRN2 and Δ2 deletion mutant expressed in the presence or not of tunicamycin. The removal of the carbohydrate chains induces a considerable reduction of the molecular weight of the molecules. P values were obtained with the ANOVA one way analysis of variance and graphs represent the mean ± SD obtained from at least three experiments.

Given that we had demonstrated that the angiostatic activity of MMRN2 relies, at least in part, on its ability to sequester the 165 isoform of VEGF-A, we verified if the deletion mutants retained the binding capability. As assessed by solid phase analyses, both the Δ1 and Δ2 mutants were able to interact to VEGF-A_165_, as opposed to the Δ3 and Δ4 mutants (Figure 3A). Thus, for the subsequent experiments, we decided to employ only the Δ2 mutant, the shortest fragment resembling the function of the entire molecule. The interaction of VEGF-A_165_ with the Δ2 deletion mutant was further confirmed by BIAcore analysis (Figure 3B). However, the kD of the interaction (kD = 4.3 × 10^−7^M), lower than that previously obtained with the whole molecule (kD = 5 × 10^−8^ M), was likely underestimated due to unstable immobilization of the mutant to the chip. Given that MMRN2 is a glycosylated molecule, we wondered if it could sequester VEGF-A through the protein core or the carbohydrate chains. To address this question, we first performed solid phase binding studies in the presence of heparin. As shown in Figure 3C, heparin completely abolished the interaction of VEGF-A_165_ with MMRN2. We next removed the sugar chains and found that the interaction with VEGF-A_165_was significantly impaired, despite the binding was not completely abolished (Figure 3D). A complete abrogation of the interaction was obtained when the removal of the carbohydrate chains was achieved under denaturing conditions. However, we could not exclude the possibility that this effect depended on the lack of proper protein folding. Similar results were obtained using the entire molecule and the Δ2 deletion mutant synthetized in the presence of tunicamycin to preclude protein glycosylation (Figure 3E). The Western blot analysis indicated that tunicamycin was effective in preventing the glycosylation but also induced a partial degradation of both recombinant molecules (Figure 3F). Thus a contribution of the protein core to the interaction with VEGF-A_165_ could not be ruled out.

**Figure 4:**
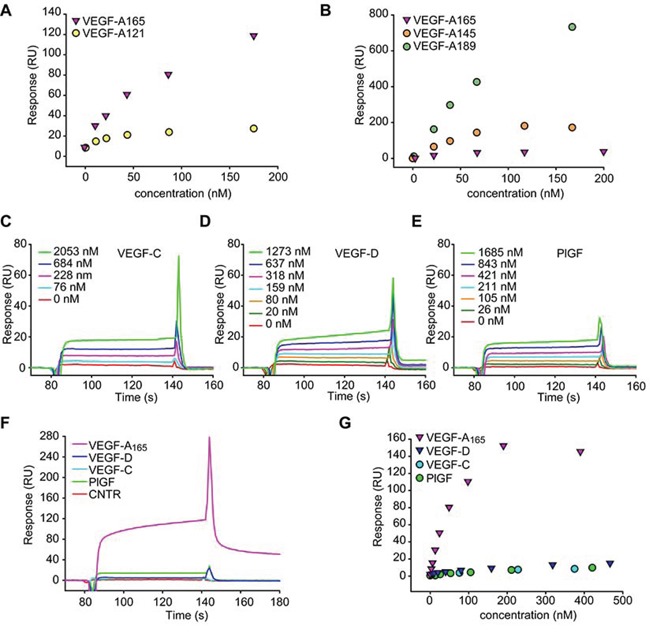
MMRN2 specifically binds to VEGF-A **A.** Dose response plot of the interaction of MMRN2 with VEGF-A_165_ and VEGF-A_121_, as obtained by surface plasmon resonance. **B.** Dose response plot of the interaction of MMRN2 with VEGF-A_165,_ VEGF-A_145_ and VEGF-A_189_, as obtained by surface plasmon resonance. **C, D, E.** Sensograms reporting the binding of VEGF-C, VEGF-D and PlGF-1, as assessed by surface plasmon resonance. **F.** Sensogram of the comparison of the binding of VEGF-A_165_, VEGF-C, VEGF-D and PlGF-1 at the concentration of 200nM to MMRN2 as assessed by surface plasmon resonance. **G.** Dose response plot of the interaction of MMRN2 with VEGF-A_165_, VEGF-C, VEGF-D and PlGF-1 as obtained by surface plasmon resonance. All experiments were repeated at least three times.

To investigate on the relative contribution of the carbohydrate chains versus the protein core, we assessed the interaction of MMRN2 with different VEGF-A isoforms, including VEGF-A_121_ which lacks the heparin binding domain. As shown in Figure 4A, the VEGF-A_121_ isoform retained the capability to bind to MMRN2 (kD = 2.0 × 10^−7^ M), despite the interaction was much lower compared to that of VEGF-A_165_. Moreover, specific binding was also detected with VEGF-A_145_ and VEGF-A_189_ (Figure 4B). Interestingly, both the heparin high-affinity binding isoforms displayed a good interaction with MMRN2, even higher than that observed with VEGF-A_165_ (kD = 2.7 × 10^−8^ M and kD = 3.0 × 10^−9^M, respectively).

We next verified if MMRN2 interacted specifically with VEGF-A_165_ or it could also bind other members of the family. The interaction of MMRN2 with VEGF-B_167_, VEGF-C and PlGF-1 was not detectable by solid phase analyses (Figure S2A to S2C). However, specific interaction between MMRN2 and VEGF-C, VEGF-D and PlGF-1 was detected through BIAcore analyses (Figure 4C to 4G), despite it was much lower compared to that of VEGF-A_165_ (kD = 7.5 × 10^−7^ M, kD = 6.4 × 10^−7^ M, and kD = 5.6 × 10^−7^ M). The interaction with VEGF-B_167_ could not be analyzed by plasmon resonance due to non-specific binding of the cytokine to the control flow cell.

### MMRN2 and the active mutant inhibit VEGFR2 activation and impair its redistribution to the EC membrane in response to VEGF-A

**Figure 5:**
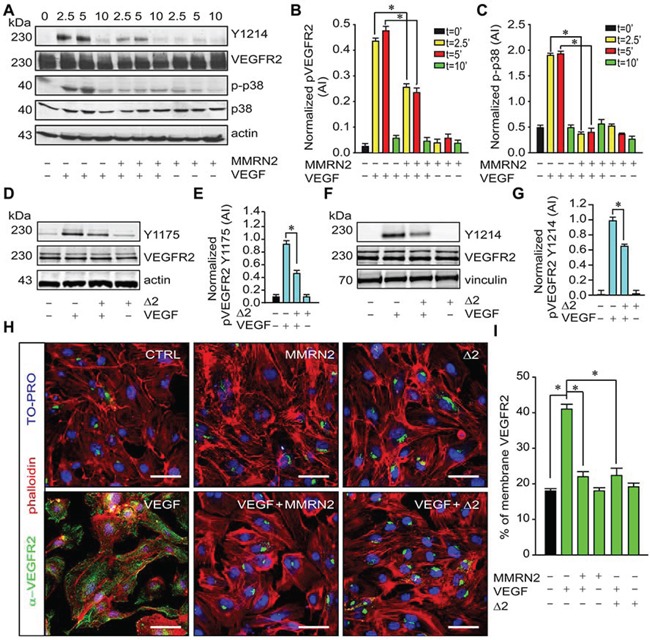
MMRN2 and the Δ2 deletion mutant impair VEFGR2 activation and its distribution at the EC surface **A.** Representative image of the Western blot analyses from the lysates of HUVEC cells challenged with MMRN2 in the presence or not of VEGF-A_165_ for different time points (2.5, 5 and 10 min as indicated). Total VEGFR2 (VEGFR2) and the phosphorylated portion at Y1214 (Y1214) were analyzed with specific antibodies along with the total (p38) and phosphorylated portion of SAPK2/p38 (p-p38). Actin was used as a normalizer of protein loading. **B.** Graph reporting the quantification, expressed in arbitrary units (AI), of VEGFR2 phosphorylation at Y1214 from the Western blot analyses reported in A, as assessed with the Image J software; (*P < 0.002). **C.** Graph reporting the quantification, expressed in arbitrary units (AI), of SAPK2/p38 phosphorylation from the Western blot analyses reported in A, as assessed with the Image J software; (*P < 0.001). **D.** Representative image of the Western blot analyses from the lysates of HUVEC cells challenged with the Δ2 deletion mutant in the presence or not of VEGF-A_165_ for 5 min. Total VEGFR2 (VEGFR2) and the phosphorylated portion at Y1175 (Y1175) were analyzed with specific antibodies. Actin was used as a normalizer of protein loading. **E.** Graph reporting the quantification, expressed in arbitrary units (AI), of VEGFR2 phosphorylation at Y1175 from the Western blot analyses reported in D; (*P = 0.008). **F.** Representative image of the Western blot analyses from the lysates of HUVEC cells challenged with the Δ2 deletion mutant in the presence or not of VEGF-A_165_ for 5 min. Total VEGFR2 (VEGFR2) and the phosphorylated portion at Y1214 (Y1214) were analyzed with specific antibodies. Vinculin was used as a normalizer of protein loading. **G.** Graph reporting the quantification, expressed in arbitrary units (AI), of VEGFR2 phosphorylation at Y1214 from the Western blot analyses reported in F; (*P = 0.003). **H.** Representative images of the immunofluorescence analyses to assess VEGFR2 distribution in HUVEC cells challenged with MMRN2 or the Δ2 deletion mutant in the presence or not of VEGF-A_165_; scale bar = 50 μm. **I.** Graph representing the quantification of the number of cells displaying VEGFR2 staining at the cell surface from the experiment reported in H, at least 10 fields each were evaluated; (*P < 0.001). P values were obtained with the ANOVA one way analysis of variance and graphs represent the mean ± SD obtained from at least three experiments.

We next analyzed the molecular mechanisms elicited by MMRN2 and verified the phosphorylation of residue Y1214 on VEGFR2, known to play an important role in SAPK2/p38 activation. MMRN2 strongly reduced Y1214 phosphorylation induced by VEGF-A_165_ as well as SAPK2/p38 activation (Figure 5A to 5C). Consistently, the Δ2 deletion mutant inhibited VEGFR2 phosphorylation at both residue Y1175 (Figure 5D and 5E) and residue Y1214 (Figure 5F and 5G). Not only MMRN2 and the Δ2 deletion mutant halted VEGFR2 phosphorylation but they also impaired the availability of the receptor at the EC membrane. In fact, HUVEC cells challenged with either of the recombinant molecule displayed a decreased VEGFR2 staining at the cell surface in response to VEGF-A_165_ treatment (Figure 5H and 5I).

### *In vivo* angiostatic and anti-tumoral function of MMRN2 and its active mutant

**Figure 6:**
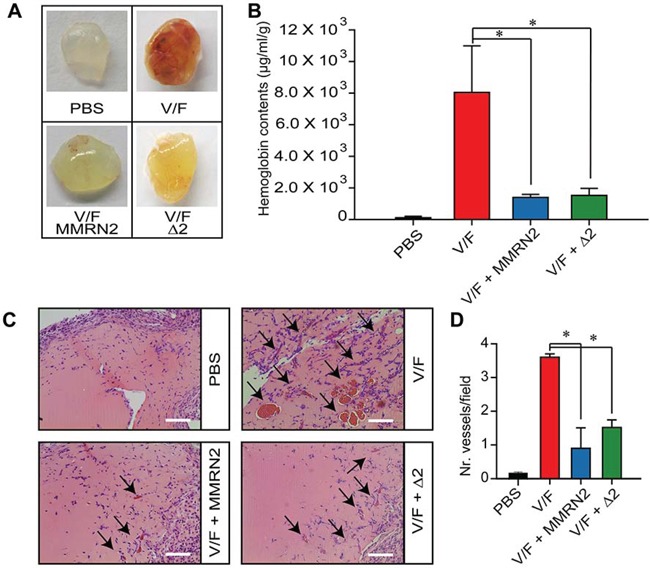
MMRN2 and the Δ2 deletion mutant impair the development of blood vessels in the *in vivo* Matrigel plug assay **A.** Representative images of the plugs explanted from BALB/c mice and challenged with 35 nM of MMRN2 or Δ2 deletion mutant, every other day for 10 days in the presence of (50 ng/ml) VEGF-A_165_ and (50 ng/ml) bFGF (V/F). Matrigel plugs were also treated with PBS as control. **B.** Graph representing the spectrophotometric evaluation of the hemoglobin content within the plugs as assessed by means of the Drabkin's reagent; (*P = 0.004). **C.** Representative images of the hematoxylin and eosin staining of the Matrigel plugs upon treatment with 35 nM of MMRN2 or Δ2 deletion mutant, every other day for 10 days in the presence of (50 ng/ml) VEGF-A_165_ and (50 ng/ml) bFGF (V/F). The newly formed vessels within the plugs are indicated by an arrow; scale bar = 100 μm. **D.** Graph representing the evaluation of the number of vessels within the plugs as assessed by counting on at least 10 fields for each point; (*P < 0.001). P values were obtained with the ANOVA one way analysis of variance and graphs represent the mean ± SD obtained from at least three experiments.

The effect of MMRN2 and of the active Δ2 deletion mutant was analyzed *in vivo*. As shown in Figure 6A and 6B, both the entire molecule and the active mutant reduced the hemoglobin content within Matrigel plugs implanted in mice. The angiostatic function was further confirmed by hematoxylin and eosin staining performed on the plugs’ sections (Figure 6C and 6D).

**Figure 7:**
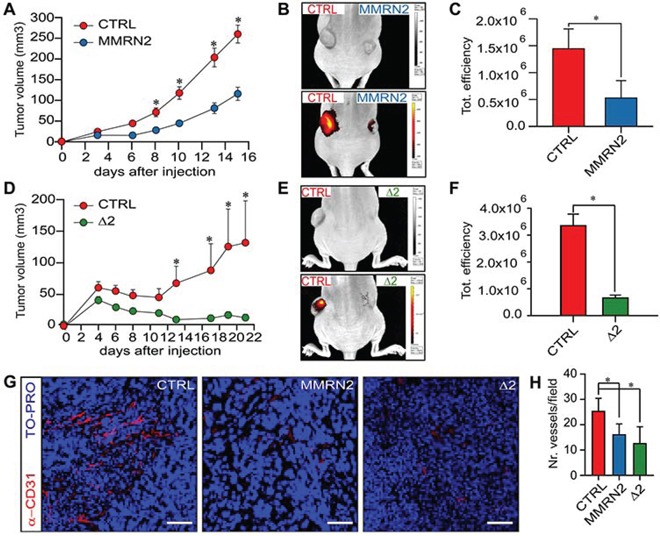
The over-expression of MMRN2 and Δ2 deletion mutant is associated with an impaired intratumoral vascularization and decreased tumor growth **A.** Graph reporting the measurements of the tumor volumes following the injection of mock-transfected HT1080 cells (CTRL, left flank) or MMRN2 over-expressing cells (MMRN2, right flank), as evaluated by means of a caliper; (*P < 0.001). **B.** Representative image of the *in vivo* Imaging analyses following injection of AngioSense® 750EX in nude mice carrying control tumors (CTRL, left flank) or MMRN2 over-expressing tumors (MMRN2, right flank). Top image mouse photograph showing the decreased tumor growth of MMRN2-ectopically expressing tumors, bottom image overlay of the photograph with the fluorescent signal of the AngioSense® 750EX probe. **C.** Graph reporting the analysis of the fluorescent signals from mock (CTRL) and MMRN2 over-expressing tumors, as assessed with the dedicated software of the IVIS® Lumina instrument; (*P = 0.03). **D.** Graph reporting the measurements of the tumor volumes following the injection of mock-transfected HT1080 cells (CTRL, left flank) or Δ2 deletion over-expressing cells (Δ2, right flank), as evaluated by means of a caliper; (*P < 0.001). **E.** Representative image of the *in vivo* Imaging analyses following injection of AngioSense® 750EX in nude mice carrying control tumors (CTRL, left flank) or Δ2 deletion over-expressing tumors (Δ2, right flank). Top image mouse photograph showing the decreased tumor growth of Δ2-ectopically expressing tumors, bottom image overlay of the photograph with the fluorescent signal of the AngioSense® 750EX probe. **F.** Graph reporting the analysis of the fluorescent signals from mock (CTRL) and Δ2 deletion over-expressing tumors, as assessed with the dedicated software of the IVIS® Lumina instrument; (*P = 0.004). **G.** Representative images of the immunofluorescent analyses performed on tumor sections from mock-, MMRN2- and Δ2-tumors (CTRL, MMRN2 and Δ2, respectively). Blood vessels were stained through an anti-CD31 antibody (α-CD31) and nuclei with TO-PRO; scale bar = 70 μm. **H.** Graph reporting the analysis of the number of vessels per field as assessed by counting in at least 10 fields from the mock, MMRN2 and Δ2 tumor sections; (*P < 0.001). P values from (A, C, D and F) were obtained using the Student's t-test, P values from (H) were obtained with the ANOVA one way analysis of variance and graphs represent the mean ± SD obtained from at least three experiments.

To verify if the Δ2 active fragment could affect tumor growth and tumor associated angiogenesis, we generated HT-1080 cells ectopically expressing the mutant. Both the Δ2 deletion mutant and the whole molecule strongly inhibited tumor growth (Figure 7A, 7B, 7D and 7E). The effect was likely indirect, since the proliferation and the apoptotic rate of these cells *in vitro* was not altered (Figure S3A and S3B). On these grounds, we assessed the extent of vascularization of the tumors. First, we injected the animals with AngioSense® 750EX and verified *in vivo* that both the whole molecule and the Δ2 deletion mutant strongly reduced the formation of the vessels within the tumors (Figure 7B, 7C, 7E and 7F). This finding was further confirmed by CD31 staining performed on the tumor sections (Figure 7G and 7H). Accordingly, possibly due to the increased hypoxia, the tumors over-expressing the two molecules displayed an increased expression of HIF-1α (Figure S3C).

In conclusion, in this study we identify the region of MMRN2 responsible for the binding to VEGF-A_165_, demonstrate that it partially involves the carbohydrate chains and verify the angiostatic activity both *in vitro* and *in vivo*.

## DISCUSSION

In this study we corroborated the angiostatic function of MMRN2 and identified the region responsible for these effects. Interestingly, these results are opposite to what observed with EMILIN2, another member of the EDEN family [50–52]. This effect occurred, at least in part, through the binding to VEGF-A_165_ via the region encompassed by the Δ2 fragment. The binding of VEGF-A_165_ required the presence of the carbohydrate chains, since their enzymatic removal partially halted the interaction. Accordingly, the binding to VEGF-A_145_ and VEGF-A_189_, which display higher affinity for heparin [53], was superior to that of VEGF-A_165_. Nonetheless, the protein core may be involved in the interaction since the removal of the carbohydrate chains did not completely abolish the binding. In fact, specific interaction was also detected with the VEGF-A_121_ isoform, which lacks the heparinbinding domain. Despite weaker, specific binding was also found with VEGF-C, -D and PlGF-1 and the low affinity may depend on a different arrangement of the basic residues involved in the interaction with the carbohydrate chains [54]. Thus, MMRN2 may bind many cytokines impinging on angiogenesis and affecting the tumor microenvironment through different mechanisms. Given the strong biological effects obtained with the Δ2 deletion mutant, we could assume that most of the VEGF-A_165_ is sequestered within this region. A further dissection of the functional fragment was attempted but none of the three smaller constructs generated could be synthetized (data not shown). Since this fragment is characterized by alternated coiled-coil regions (Figure S4), it is possible that the dissection prevented a proper trimerization, thus leading to protein degradation.

The binding of VEGF-A_165_ to MMRN2 and the Δ2 deletion mutant led to a decreased phosphorylation of VEGFR2 at Y1175 and also Y1214, which is known to prompt SAPK2/p38 activation [55]. In addition, we identified a further level of VEGFR2 de-activation triggered by MMRN2. This involved the intracellular confinement of the receptor in the Golgi apparatus (Figure 5H and 5I) reducing its availability at the cell surface. The binding of VEGF-A to VEGFR2 induces the exit of intracellular VEGFR2 from the Golgi apparatus en route to the plasma membrane [56]. The treatment of EC with MMRN2 or the Δ2 deletion mutant did not decrease the total levels of VEGFR2, thus the impaired recruitment of the receptor at the cell surface may strictly depend on a decreased availability of VEGF-A_165_ for receptor engagement.

In a recent publication it was shown that the blockage of CLEC14A-MMRN2 interaction inhibits sprouting angiogenesis and tumor growth [57]. These findings are in contrast with our published observations indicating that down-regulation of the MMRN2 endogenous expression increased EC migration [49]. In addition, here we demonstrated that the down-regulation of MMRN2 expression strengthened the sprouting of ECs from the spheroids (Figure 2G to 2I). It is possible that these opposite results may depend on the different molecules used to generate the matrix spheroids have been embedded in. However, given that MMRN2 is physiologically expressed along all the vessels, it is more likely that it exerts a homeostatic role, halting the sprouting of new vessels unless a strong pro-angiogenic stimulus is engaged.

To assess the role of MMRN2 and its active deletion mutant in tumor-associated angiogenesis, we employed HT1080 cells since the two molecules did not affect their proliferation or their apoptotic rate. This indicated that the strong decrease of tumor growth was due to an indirect effect, and likely depended on an impaired vascular supply. Accordingly, both the *in vivo* analyses and the examinations of the tumor sections indicated that the over-expression of MMRN2 and the Δ2 deletion mutant halted the development of tumor associated vessels. As a consequence, the increased intra-tumoral hypoxia likely induced the expression of HIF-1α, which was particularly high in the Δ2 mutant over-expressing tumors.

In conclusion, the present results provide additional evidences indicating an angiostatic role for MMRN2 and identify the region of the molecule responsible for the functional effects. Given that expression of MMRN2 is altered in a number of tumor types [49,58–60], it is conceivable that the growth of the tumors as well as the therapeutic efficacy may be significantly affected depending on the levels of MMRN2 expression.

## MATERIALS AND METHODS

### Cell cultures

Human Umbilical Vein Endothelial Cells (HUVEC) were isolated from the human umbilical cord vein as previously described [61]. Cells were cultured in M199 medium (GIBCO, Invitrogen, Milan, Italy) supplemented with 20% fetal bovine serum (FBS) (GIBCO, Invitrogen, Milan, Italy), 1% Penicillin-Streptomycin (Sigma-Aldrich, Milan, Italy), 50 mg/ml heparin (Sigma-Aldrich, Milan, Italy) and bovine brain extract (0,5%). Embryonic kidney 293-EBNA (Epstein-Barr Nuclear Antigen) cells were a gift from Rupert Timpl (Max Planck, Munich, Germany) and were cultured in Dulbecco's modified Eagle medium (DMEM) (Sigma-Aldrich, Milan, Italy) containing 10% FBS, 1% Penicillin-Streptomycin and 250 μg/ml of G418 (Sigma-Aldrich, Milan, Italy); 0.5 μg/ml of puromycin (Sigma-Aldrich, Milan, Italy) were added after transfection. The human fibrosarcoma (HT1080) cell line was obtained from American Type Culture Collection (ATCC, Manassas, VA) and cultured in DMEM containing 10% FBS, 1% Penicillin-Streptomycin and 600 μg/ml of G418 after transfection. Normal human dermal fibroblast (NHDF) cells were obtained from LONZA (Basel, Switzerland) and maintained in DMEN supplemented with 10% FBS and 1% Penicillin-Streptomycin. All cells were maintained at 37°C in a humidified 5% CO_2_ atmosphere.

### Antibodies and other reagents

The anti-histidine antibody was from Abgent (San Diego, CA, USA), the Ni-NTA agarose was from QIAGEN (Milan, Italy). The anti-MMRN2 polyclonal antibody was obtained upon immunization of a rabbit with 150 μg of a recombinant MMRN2 fragment corresponding to the N-terminal gC1q domain. The antibody was affinity purified from the rabbit serum by means of the CNBr-activated Sepharose 4B resin (Amersham, GE-Healthcare, Milan, Italy). The secondary horse radish peroxidase (hrp)-conjugated antibodies were from Amersham (GE-Healthcare, Milan, Italy). The secondary antibodies conjugated with Alexa Fluor 488, 568 and TO-PRO-3 were from Invitrogen (Milan, Italy). Recombinant human VEGF-A_165_, VEGF-A_145_ and VEGF-A_189_ proteins were from R & D systems, Inc (MN, USA) and VEGF-A_121_ fromPeprotech (London, UK). The basic FGF, VEGF-B_167_, VEGF-C, VEGF-D and PlGF-1 were from Peprotech (Rocky Hill, NJ). The anti-CD31 antibody and Matrigel were from BD Biosciences. The anti-VEGFR2 and anti phospho-VEGFR2 (Tyr1175) and (Tyr1214), the anti-p38 and anti-phospho-p38, and the anti-β-actin antibodies were from Cell Signaling Technology Inc. (Danvers, MA, USA). The anti-VEGF-A antibody was from Sigma-Aldrich (Milan, Italy). Anti-VEGF-B, anti-VEGF-C, anti-VEGF-D and anti-PlGF-1 were from Santa Cruz Biotechology Inc. (California, USA). The *in situ* Cell Death Detection Fluorescein Kit was purchased from Roche Diagnostics S.p.a. (Milan, Italy). Cytodex 3 microcarriers were from GE Healthcare Life Sciences (Milan, Italy). Drabkin reagent kit and Tunicamycin were purchased from Sigma-Aldrich (Milan, Italy); AngioSense® 750EX fluorescent imaging agent was from PerkinElmer (Waltham, Massachusetts).

### DNA constructs and Real Time PCR analyses

The following MMRN2 deletion mutants were created: Δ1 (aa residues 24 to 474), Δ2 (aa residues 137 to 336), Δ3 (aa residues 348 to 683) and Δ4 (aa residues 674 to 949). Δ1, Δ2 and Δ3 fragments were amplified from the full length molecule and cloned into the pCEP-Pu vector containing the BM40 signal peptide sequence using the following oligonucleotides: Δ1: 5′-ctagctagcccatcatcaccatcaccatgcttccagtactagcctc-3′ containing the NheI site and His sequence; 5′-atagtttagcggccgctcagaggttgagctccaggag-3′ containing the NotI site; Δ2: 5′-ctagctagccccatcatcaccatcaccatccaatccctgagcctgca-3′ containing the NheI site and His sequence; 5′-atagtttagcggccgctcatttggtgtccacatcggc-3′ containing the NotI site; Δ3: 5′-gcaacagctgtccatcatcaccatcaccatgggaccaatggcagtctggtg-3′ containing the pshAI site and the His sequence; 5′-cgggatccgtcgtggctgggctccag-3′ containing the BamHI site; Δ4: 5′-gctagcccatcatcaccatcaccatCCGGCAGAGCACCTGGAG-3′ containing the NheI site and His sequence; 5′-atagtttagcggccgctcaTCAGGTCTTAAACATCAGG-3′ containing the NotI site. In addition, the MMRN2 or Δ2 cDNA were sub-cloned into pcDNA3.1/Myc-His vector by *Hind* III and *Bam* HI restriction. RNA was extracted from tumor frozen sections with the Trizol reagent (Invitrogen, Milan, Italy)., and reverse transcription performed using AMV-RT and exanucleotides (Promega, Milan, Italy). Real-time PCRs were carried out using the iQ™ SYBR® Green Supermix (Bio-Rad, Hercules, CA, USA) using the following oligonucleotides: GAPDH 5′-GAGAGACCCTCACTGCTG-3′, 5′-GATGGTACATGACAAGGTGC-3′; HIF-1α 5′-CAGAGCAGGAAAAGGAGTCA-3′, 5′-AGTAGCTGCATGATCGTCTG-3′; The primer efficiency was ∼100%, thus the comparative Ct method (2^−ΔΔCt^) was applied for the analyses.

### Cell transfection, expression and purification of recombinant proteins

293-EBNA cells were transfected by electroporation with the different pCEP-Pu constructs and selected in the presence of 0,5 μg/ml of puromycin and 250 μg/ml of G418. Positive clones were isolated and the expression analyzed by Western blotting. Confluent 293-EBNA cells were then incubated in serum-free medium for 48 hours, the media were collected and equilibrated with a buffer containing 50 mM NaH_2_PO_4_, 150 mM NaCl, 10 mM imidazole. The proteins were purified by means of the Ni-NTA resin and eluted with the elution buffer (50 mM NaH_2_PO_4_, 300 mM NaCl, 250 mM imidazole). The different fractions were analyzed by SDS-PAGE followed by Coomassie blue staining. Protein fractions were then dialyzed against PBS and concentrated using polyethylene glycol (PEG). In addition, HT1080 cells were stably transfected by electroporation with the pcDNA constructs and selected in the presence of 600 μg/ml of G418.

### Scratch test and cell migration assays

For the scratch test HUVECs were seeded in a 24-multiwell dish and allowed to grow until they reached confluency. Cells were then starved overnight and the day after a scratch wound across each well was made using a sterile pipet tip. Cells were washed to remove any loosely held cells and then incubated with medium containing 0,5 % serum in the presence of 5 μg/ml of purified MMRN2 or the equimolar concentrations (35nM) of Δ1, Δ2, Δ3 and Δ4 purified fragments, or type I collagen as a control. The open gap was then inspected over time with the microscope. Time course analysis was carried out by means of the LEICA AF6000 Imaging System (LEICA, Wetzlar, Germany).

For the motility assay the transwell membranes carrying 8 μm pores were coated on the upper side with 5 μg/ml of MMRN2 or the equimolar concentrations (35nM) of Δ1 or Δ2 or Δ3 or Δ4 fragments in the presence of 0,1M bicarbonate buffer pH 9,6 at 4°C overnight. Type I collagen was used as control. The next day the membranes were saturated with 1% BSA in PBS for 1 hour at room temperature. 1 × 10^5^ HUVEC cells were placed on the top layer of the permeable membrane in serum free M199 medium containing 0,1% BSA. In the bottom chamber VEGF-A was added to the medium as migratory stimulus at the concentration of 25 ng/ml. After 6 hours of migration cells were stained with Crystal violet for 30 minutes and counted.

### Cell viability and proliferation assays

HUVEC cells were incubated with 35 nM of MMRN2 or PBS for 24, 48 and 72 hours; in alternative the cells were challenged with MMRN2, collagen type I and the various deletion mutants for 48 hours and cell viability was analyzed. The MTT (3-(4,5-Dimethylthiazol-2-yl)-2,5-diphenyltetrazolium bromide, a tetrazole) reagent was added to the cells at a final concentration of 0,3 mg/ml and incubated for 4 hours at 37°C in complete medium. The medium was discarded and the crystals solubilized with dimethyl sulfoxide (DMSO). The reduced form of the colorimetric substrate was then quantified at the spectrophotometer at 560 nm. Cell proliferation was assessed by culturing the mock and Δ2-transfected HT1080 cells in 96-well plates for 24, 48, 72 and 96 hours. Cells were stained with the Trypan blue solution (Sigma-Aldrich, Milan, Italy) and counted using a hematocytometer.

### TUNEL assays

The apoptotic rate was evaluated using the “*in situ* cell death detection kit, fluorescein” (Roche Diagnostics S.p.a, Milan, Italy) upon treatment of HUVECs with 5 μg/mL recombinant MMRN2 or with the equimolar concentrations (35nM) of Δ1, Δ2, Δ3 and Δ4 purified fragments for 48 hours, and the assay performed according to the manufacturer's instructions. Lipopolysaccharides from Salmonella enteric Serotype enteritidis (LPS, Sigma-Aldrich, Milan, Italy) at the concentration of 100 ng/ml was used to induce HUVEC cell apoptosis. Briefly, the cells were fixed in 4% PFA for 20 minutes at room temperature, permeabilized for 2 minutes in freshly prepared permeabilization solution (sodium citrate 0,1%, Triton X-100 0,1%) at 4°C and incubated with the properly diluted enzyme solution for 1 hour at 37°C in humidified conditions. The cells were mounted in Fluoroshield™ with DAPI (Sigma-Aldrich, Milan, Italy) and positive cells counted using a fluorescence microscope equipped with a 63X objective. The same protocol was used to score the apoptotic rate in HT1080 cells stably expressing the Δ2 fragment of MMRN2.

### Matrigel tube formation assay

The growth factor reduced Matrigel TM Matrix (BD Biosciences) was thawed at 4°C overnight; 40 μl were quickly added to each well of a 96-multiwell dish using cold pipettes and was allowed to solidify for 30 min at 37°C. Once solid, 1 × 10^4^ HUVEC cells were resuspended in medium containing 0,5% serum and 5 μg/ml of purified MMRN2 or the equimolar concentrations (35nM) of Δ1, Δ2, Δ3 or Δ4 purified fragments, or type I collagen as a control and then seeded in each well. Time-course analyses was carried out for 12 hours by means of LEICA AF6000 Imaging System. Tube formation analysis was assessed with the Wimasis software.

### 3D *in vitro* angiogenesis assay

The 3D *in vitro* spheroid based angiogenesis tests were performed as previously described [62]. Briefly, 4 × 10^2^ HUVEC cells per cytodex microcarrier were employed. ECs were incubated with the beads for 4 hours at 37°C, shaking every 20 minutes. After the incubation time, the coated beads were transferred into a flask containing complete medium and were incubated overnight at 37°C. The next day the coated beads were embedded into a fibrin gel with or without 35nM of MMRN2 or the Δ1, Δ2, Δ3 and Δ4 purified fragments. To provide the required soluble factors to promote EC sprouting, NHDF cells were layered on top of the gel after resuspension in medium containing the purified fragments, in combination or not with VEGF-A (50 ng/ml). After 7 days spheroids were fixed with 4% (w/v) paraformaldehyde for 15 minutes at room temperature and pictures were captured and analyzed by Image J software.

### ELISA tests

For the analysis of the binding of MMRN2 or Δ1, Δ2, Δ3 and Δ4 fragments with VEGF-A, 0.5 μg of the recombinant MMRN2 or the deletion mutants were used to coat the plates and BSA was used as a control. The wells were blocked with 2% BSA in PBS for 1 hour at room temperature and incubated with soluble VEGF-A (100 ng/well) in 0,2% BSA in PBS for 1 hour at 37°C. In other sets of experiments, the MMRN2-coated wells were incubated with soluble VEGF-B_167_, VEGF-C, VEGF-D or PlGF-1. Binding was verified using the specific anti-cytokine antibodies; the ABTS substrate was added and absorbance at 405 nm detected with a spectrophotometer (TECAN, Milan, Italy).

### Surface plasmon resonance tests

The affinity measurements were performed using a Biacore X100 biosensor (GE Healthcare) on a carboxymethyldextran-coated sensor chip (CM5) as previously described [49]. The purified MMRN2 (20 ng/μl) or the Δ2 fragment (80 ng/μl) in Na acetate pH=4 were immobilized using amine coupling to a density of 3150 and 1770 resonance units (RU), respectively. VEGF-A_165_ and VEGF-A_121_, VEGF-B_167_, VEGF-C, VEGF-D and PlGF-1 were diluted in HBS-EP buffer (GE Healthcare) at different concentrations and injected over the sensor chip at a flow rate of 30 μL/min, with 60 seconds of analyte contact over the surface. In other sets of experiments when analyzing the interaction with VEGF-A_145_ and VEGF-A_189_, a NaCl concentration of 300 mM instead of 150 mM was employed. The kinetic parameters and dissociation constants (*k*D) were then determined using the BIAevaluation software.

### Deglycosylation and Tunicamicyn treatments

The cleavage of the MMRN2 carbohydrate chains was performed using the Protein Deglycosylation Mix purchased from New England Biolabs (Beverly, MA) according to manufacturer's instructions. Briefly, 50 μg of purified MMRN2 were incubated with deglycosylation mix under non denaturing or denaturing conditions for 4 hours at 37°C. The deglycosylated protein was used for the analysis of the MMRN2/VEGF-A interaction by ELISA test, as previously described.

For the inhibition of N-linked glycosylation, 293-EBNA cell stably expressing MMRN2 or Δ2 fragment were treated or not with 5 μg/ml of Tunicamycin every 2 hours for 24 hours in serum free medium. The non-glycosylated purified proteins were analyzed by Western blotting and used in the solid phase analysis to evaluate the binding with VEGF-A.

### Preparation of cell lysates and Western blot analysis

For the phosphorylation studies, HUVEC cells were treated with VEGF-A (15ng/ml) with or without MMRN2 (5 μg/ml) or the equimolar concentration (35nM) of the Δ2 purified fragment for different times. The cells were then lysed in cold buffer (1mM CaCl2, 1mM MgCl2, 15mM Tris-HCl pH 7.2, 150mM NaCl, 1% TrytonX100, 0,1% SDS, 0,1% Na Deoxycholate) containing 25 mM NaF, 1 mM DTT, 1 mM Na3VO4 and the protease inhibitors cocktail (Roche). For the Western blot analyses proteins were resolved in 4–20% Criterion Precast Gels (Bio-Rad Laboratories) and transferred onto Hybond-ECL nitrocellulose membranes (Amersham, GE-Healthcare). Membranes were blocked with 5% BSA in TBS-T (100mM Tris-HCl pH 7.5, 0,9% NaCl, 0,1% Tween 20) and probed with the appropriate antibodies. The blots were finally developed using ECL (Western blotting detection, Amersham Biosciences) and exposed to X-ray films or acquired using the ChemiDoc Touch Imaging System (BIO RAD, Hercules, CA, USA). Alternatively the Odyssey infrared imaging system was used (Li-COR Biosciences, Lincoln, NE, USA).

### Matrigel plug angiogenesis assay

Ten female BALB/c (Harlan S.r.l, Milan, Italy) mice were subcutaneously injected (0.5 ml/flank) with highly-concentrated (18 mg/ml) Matrigel containing PBS (five left flanks) or 50 ng/ml of b-FGF and VEGF-A (five left flanks) or 50ng/ml of b-FGF and VEGF-A with 35 nM of MMRN2 or Δ2 (10 right flanks). Every other day the growth factors and the recombinant proteins were re-injected into the plugs into a final volume of 100 μl. After 10 days, the mice were sacrificed and the Matrigel plugs were excised. The plugs were divided in two parts, one half was fixed with formalin overnight, embedded in paraffin and sectioned onto slides stained with hematoxylin and eosin for histological observation. The remaining plugs were homogenized and the hemoglobin content was evaluated using the Drabkin reagent kit (Sigma-Aldrich, Milan, Italy), as previously described [63].

### *In vivo* tumor growth

Twenty female athymic nude mice (Harlan S.r.l, Milan, Italy) were injected with 1.5 × 10^6^ of HT1080 cells stably transfected with pcDNA3.1 vector carrying the MMRN2 or Δ2 coding sequence or with the empty vector. The left flanks of each mouse were injected with control cells, while the right flanks with cells expressing MMRN2 or Δ2. Tumor growth was monitored over time and tumor size measured with a caliper. The tumor volumes were calculated with the following formula: (Pxlengthxwidth^2^)/6. Tumor vascularity was imaged using AngioSense® 750EX (PerkinElmer). Anesthetized mice were retro-orbital injected with 2 nmol of AngioSense® 750EX in 100 μL of PBS and the fluorescence signal detected after 24 hour by IVIS Lumina instrument (Perkinelmer, Walthman, MA, USA). The mice were sacrificed and the tumors excised for immunofluorescence analysis. All the *in vivo* studies were approved by the Institutional Ethics Committee.

### Immunofluorescence analysis of ECs and tumors sections

HUVECs were grown on cover glass slides placed in a 24 multi-well plate, treated with VEGF-A (10 ng/ml) and MMRN2 (5 μg/ml) or equimolar concentration (35nM) of Δ2 for 20 min at 37°C and then fixed with 4% (w/v) paraformaldehyde for 15 minutes at room temperature. The cells were permeabilized with a PBS solution containing 1% BSA, 0,2% TRITON X-100 for 5 minutes at room temperature, saturated with blocking buffer (PBS-2% BSA) for 1 hour and incubated overnight at 4°C with the α-VEGFR2 antibody. Next the actin cytoskeleton and the nuclei were stained for 1 hour at room temperature with phalloidin and TO-PRO3, respectively. Slides were finally mounted in Mowiol containing 2,5% (w/v) of 1,4-diazabicyclo-(2,2,2)-octane (DABCO). The number of cells displaying VEGFR2 staining at the cell surface was evaluated by counting.

For the immunofluorescence analyses, tumors were included in the Optimal Cutting Temperature compound (OCT) and frozen. For microvessel density analysis, 7 μm thick sections were obtained and stained with anti-mouse CD31. Images were acquired with a Leica TCS SP2 confocal system (Leica Microsystems Heidelberg, Mannheim, Germany), using the Leica Confocal Software (LCS). Vessels density was assessed by counting.

### Statistical analyses

Statistical analyses were performed using the Sigma Plot software. Student's t-test for unpaired data was used to assess the probability of significant differences between two groups; for more than two groups, the ANOVA 1-way analysis of variance was used, according to the Bonferroni method. Results with p ≤ 0.05 were considered significant.

## SUPPLEMENTARY FIGURES



## References

[1] Risau W (1997). Mechanisms of angiogenesis. Nature.

[2] Carmeliet P (2000). Mechanisms of angiogenesis and arteriogenesis. Nat Med.

[3] Carmeliet P (2003). Angiogenesis in health and disease. Nat Med.

[4] Hanahan D, Weinberg RA (2011). Hallmarks of cancer: the next generation. Cell.

[5] Folkman J (1971). Tumor angiogenesis: therapeutic implications. N Engl J Med.

[6] Ebos JM, Lee CR, Cruz-Munoz W, Bjarnason GA, Christensen JG, Kerbel RS (2009). Accelerated metastasis after short-term treatment with a potent inhibitor of tumor angiogenesis. Cancer Cell.

[7] Carmeliet P, Jain RK (2011). Molecular mechanisms and clinical applications of angiogenesis. Nature.

[8] Cascone T, Heymach JV (2012). Targeting the angiopoietin/Tie2 pathway: cutting tumor vessels with a double-edged sword?. J Clin Oncol.

[9] Chung AS, Lee J, Ferrara N (2010). Targeting the tumour vasculature: insights from physiological angiogenesis. Nat Rev Cancer.

[10] Ohtsu A, Shah MA, Van CE, Rha SY, Sawaki A, Park SR, Lim HY, Yamada Y, Wu J, Langer B, Starnawski M, Kang YK (2011). Bevacizumab in combination with chemotherapy as first-line therapy in advanced gastric cancer: a randomized, double-blind, placebo-controlled phase III study. J Clin Oncol.

[11] Okines A, Cunningham D (2009). Current perspective: bevacizumab in colorectal cancer—a time for reappraisal?. Eur J Cancer.

[12] Paez-Ribes M, Allen E, Hudock J, Takeda T, Okuyama H, Vinals F, Inoue M, Bergers G, Hanahan D, Casanovas O (2009). Antiangiogenic therapy elicits malignant progression of tumors to increased local invasion and distant metastasis. Cancer Cell.

[13] Ferrara N (2005). VEGF as a therapeutic target in cancer. Oncology.

[14] Jain RK (2005). Normalization of tumor vasculature: an emerging concept in antiangiogenic therapy. Science.

[15] Sorensen AG, Emblem KE, Polaskova P, Jennings D, Kim H, Ancukiewicz M, Wang M, Wen PY, Ivy P, Batchelor TT, Jain RK (2012). Increased survival of glioblastoma patients who respond to antiangiogenic therapy with elevated blood perfusion. Cancer Res.

[16] Emblem KE, Mouridsen K, Bjornerud A, Farrar CT, Jennings D, Borra RJ, Wen PY, Ivy P, Batchelor TT, Rosen BR, Jain RK, Sorensen AG (2013). Vessel architectural imaging identifies cancer patient responders to anti-angiogenic therapy. Nat Med.

[17] Jain RK (2014). Antiangiogenesis strategies revisited: from starving tumors to alleviating hypoxia. Cancer Cell.

[18] Maes H, Kuchnio A, Peric A, Moens S, Nys K, De-áBock K, Quaegebeur A, Schoors S, Georgiadou M, Wouters J, Vinckier S, Vankelecom H, Garmyn M, Vion ACm, Radtke F, Boulanger C, Gerhardt H, Dejana E, Dewerchin M, Ghesquiere B, Annaert W, Agostinis P, Carmeliet P (2014). Tumor Vessel Normalization by Chloroquine Independent of Autophagy. Cancer Cell.

[19] Wong PP, Demircioglu F, Ghazaly E, Alrawashdeh W, Stratford MR, Scudamore CL, Cereser B, Crnogorac-Jurcevic T, McDonald S, Elia G, Hagemann T, Kocher HM, Hodivala-Dilke KM (2015). Dual-Action Combination Therapy Enhances Angiogenesis while Reducing Tumor Growth and Spread. Cancer Cell.

[20] Naldini A, Carraro F (2005). Role of inflammatory mediators in angiogenesis. Curr Drug Targets Inflamm Allergy.

[21] Ronca R, Giacomini A, Di SE, Coltrini D, Pagano K, Ragona L, Matarazzo S, Rezzola S, Maiolo D, Torrella R, Moroni E, Mazzieri R, Escobar G, Mor M, Colombo G, Presta M (2015). Long-Pentraxin 3 Derivative as a Small-Molecule FGF Trap for Cancer Therapy. Cancer Cell.

[22] Tarallo V, De Falco S (2015). The vascular endothelial growth factors and receptors family: Up to now the only target for anti-angiogenesis therapy. Int J Biochem Cell Biol.

[23] Ingber DE, Folkman J (1989). How does extracellular matrix control capillary morphogenesis?. Cell.

[24] Slevin M, Krupinski J, Gaffney J, Matou S, West D, Delisser H, Savani RC, Kumar S (2007). Hyaluronan-mediated angiogenesis in vascular disease: Uncovering RHAMM and CD44 receptor signaling pathways. Matrix Biol.

[25] Neve A, Cantatore FP, Maruotti N, Corrado A, Ribatti D (2014). Extracellular matrix modulates angiogenesis in physiological and pathological conditions. Biomed Res Int.

[26] Senger DR, Davis GE (2011). Angiogenesis. Cold Spring Harb Perspect Biol.

[27] Cheresh DA, Stupack DG (2008). Regulation of angiogenesis: apoptotic cues from the ECM. Oncogene.

[28] Nyberg P, Salo T, Kalluri R (2008). Tumor microenvironment and angiogenesis. Front Biosci.

[29] Sweeney SM, DiLullo G, Slater SJ, Martinez J, Iozzo RV, Lauer-Fields JL, Fields GB, Antonio JDS (2003). Angiogenesis in Collagen I Requires alpha2beta1 Ligation of a GFP*GER Sequence and Possibly p38 MAPK Activation and Focal Adhesion Disassembly. J Biol Chem.

[30] Mammoto T, Jiang A, Jiang E, Panigrahy D, Kieran MW, Mammoto A (2013). Role of Collagen Matrix in Tumor Angiogenesis and Glioblastoma Multiforme Progression. Am J Pathol.

[31] Nicosia RF, Bonanno E, Smith M (1993). Fibronectin promotes the elongation of microvessels during angiogenesis *in vitro*. J Cell Physiol.

[32] Yi M, Ruoslahti E (2001). A fibronectin fragment inhibits tumor growth, angiogenesis, and metastasis. Proc Natl Acad Sci.

[33] Li R, Luo M, Ren M, Chen N, Xia J, Deng X, Zeng M, Yan K, Luo T, Wu J (2014). Vitronectin Regulation of Vascular Endothelial Growth Factor-Mediated Angiogenesis. J Vasc Res.

[34] Simon-Assmann P, Orend G, Mammadova-Bach E, Spenle C, Lefebvre O (2011). Role of laminins in physiological and pathological angiogenesis. Int J Dev Biol.

[35] Lawler PR, Lawler J (2012). Molecular Basis for the Regulation of Angiogenesis by Thrombospondin-1 and -2. Cold Spring Harb Perspect Med.

[36] Jendraschak E, Helene Sage E (1996). Regulation of angiogenesis by SPARC and angiostatin: implications for tumor cell biology. Semin Cancer Biol.

[37] Aviezer D, Hecht D, Safran M, Eisinger M, David G, Yayon A (1994). Perlecan, basal lamina proteoglycan, promotes basic fibroblast growth factor-receptor binding, mitogenesis, and angiogenesis. Cell.

[38] Jarvelainen H, Sainio A, Wight TN (2015). Pivotal role for decorin in angiogenesis. Matrix Biol.

[39] Marneros AG, Olsen BR (2005). Physiological role of collagen XVIII and endostatin. FASEB J.

[40] Kamphaus GD, Colorado PC, Panka DJ, Hopfer H, Ramchandran R, Torre A, Maeshima Y, Mier JW, Sukhatme VP, Kalluri R (2000). Canstatin, a Novel Matrix-derived Inhibitor of Angiogenesis and Tumor Growth. J Biol Chem.

[41] Maeshima Y, Sudhakar A, Lively JC, Ueki K, Kharbanda S, Kahn CR, Sonenberg N, Hynes RO, Kalluri R (2002). Tumstatin, an Endothelial Cell-Specific Inhibitor of Protein Synthesis. Science.

[42] Mongiat M, Sweeney SM, San Antonio JD, Fu J, Iozzo RV (2003). Endorepellin, a novel inhibitor of angiogenesis derived from the C terminus of perlecan. J Biol Chem.

[43] Willis CD, Poluzzi C, Mongiat M, Iozzo RV (2013). Endorepellin laminin-like globular 1/2 domains bind Ig3–5 of vascular endothelial growth factor (VEGF) receptor 2 and block pro-angiogenic signaling by VEGFA in endothelial cells. FEBS J.

[44] Braghetta P, Ferrari A, De GP, Zanetti M, Volpin D, Bonaldo P, Bressan GM (2004). Overlapping, complementary and site-specific expression pattern of genes of the EMILIN/Multimerin family. Matrix Biol.

[45] Christian S, Ahorn H, Novatchkova M, Garin-Chesa P, Park JE, Weber G, Eisenhaber F, Rettig WJ, Lenter MC (2001). Molecular cloning and characterization of EndoGlyx-1, an EMILIN-like multisubunit glycoprotein of vascular endothelium. J Biol Chem.

[46] Sanz-Moncasi MP, Garin-Chesa P, Stockert E, Jaffe EA, Old LJ, Rettig WJ (1994). Identification of a high molecular weight endothelial cell surface glycoprotein, endoGlyx-1, in normal and tumor blood vessels. Lab Invest.

[47] Huber MA, Kraut N, Schweifer N, Dolznig H, Peter RU, Schubert RD, Scharffetter-Kochanek K, Pehamberger H, Garin-Chesa P (2006). Expression of stromal cell markers in distinct compartments of human skin cancers. J Cutan Pathol.

[48] Koperek O, Scheuba C, Puri C, Birner P, Haslinger C, Rettig W, Niederle B, Kaserer K, Garin CP (2007). Molecular characterization of the desmoplastic tumor stroma in medullary thyroid carcinoma. Int J Oncol.

[49] Lorenzon E, Colladel R, Andreuzzi E, Marastoni S, Todaro F, Schiappacassi M, Ligresti G, Colombatti A, Mongiat M (2012). MULTIMERIN2 impairs tumor angiogenesis and growth by interfering with VEGF-A/VEGFR2 pathway. Oncogene.

[50] Mongiat M, Ligresti G, Marastoni S, Lorenzon E, Doliana R, Colombatti A (2007). Regulation of the extrinsic apoptotic pathway by the extracellular matrix glycoprotein EMILIN2. Mol Cell Biol.

[51] Marastoni S, Andreuzzi E, Paulitti A, Colladel R, Pellicani R, Todaro F, Schiavinato A, Bonaldo P, Colombatti A, Mongiat M (2014). EMILIN2 down-modulates the Wnt signalling pathway and suppresses breast cancer cell growth and migration. J Pathol.

[52] Mongiat M, Marastoni S, Ligresti G, Lorenzon E, Schiappacassi M, Perris R, Frustaci S, Colombatti A (2010). The extracellular matrix glycoprotein elastin microfibril interface located protein 2: a dual role in the tumor microenvironment. Neoplasia.

[53] Vempati P, Popel AS, Mac GF (2014). Extracellular regulation of VEGF: isoforms, proteolysis, and vascular patterning. Cytokine Growth Factor Rev.

[54] Chiodelli P, Bugatti A, Urbinati C, Rusnati M (2015). Heparin/Heparan sulfate proteoglycans glycomic interactome in angiogenesis: biological implications and therapeutical use. Molecules.

[55] Lamalice L, Houle F, Huot J (2006). Phosphorylation of Tyr1214 within VEGFR-2 triggers the recruitment of Nck and activation of Fyn leading to SAPK2/p38 activation and endothelial cell migration in response to VEGF. J Biol Chem.

[56] Manickam V, Tiwari A, Jung JJ, Bhattacharya R, Goel A, Mukhopadhyay D, Choudhury A (2011). Regulation of vascular endothelial growth factor receptor 2 trafficking and angiogenesis by Golgi localized t-SNARE syntaxin 6. Blood.

[57] Noy PJ, Lodhia P, Khan K, Zhuang X, Ward DG, Verissimo AR, Bacon A, Bicknell R (2015). Blocking CLEC14A-MMRN2 binding inhibits sprouting angiogenesis and tumour growth. Oncogene.

[58] Soltermann A, Ossola R, Kilgus-Hawelski S, von EA, Suter T, Aebersold R, Moch H (2008). N-glycoprotein profiling of lung adenocarcinoma pleural effusions by shotgun proteomics. Cancer.

[59] Shield-Artin KL, Bailey MJ, Oliva K, Liovic AK, Barker G, Dellios NL, Reisman S, Ayhan M, Rice GE (2012). Identification of ovarian cancer-associated proteins in symptomatic women: a novel method for semi-quantitative plasma proteomics. Proteomics Clin Appl.

[60] Zanivan S, Maione F, Hein MY, Hernandez-Fernaud JR, Ostasiewicz P, Giraudo E, Mann M (2013). SILAC-Based Proteomics of Human Primary Endothelial Cell Morphogenesis Unveils Tumor Angiogenic Markers. Mol Cell Proteomics.

[61] Jaffe EA, Nachman RL, Becker CG, Minick CR (1973). Culture of human endothelial cells derived from umbilical veins. Identification by morphologic and immunologic criteria. J Clin Invest.

[62] Nakatsu MN, Davis J, Hughes CC (2007). Optimized fibrin gel bead assay for the study of angiogenesis. J Vis Exp.

[63] Kang K, Lim JS (2012). Induction of functional changes of dendritic cells by silica nanoparticles. Immune Netw.

