# Supplementary material for: MULTIMERIN2 binds VEGF-A primarily via the carbohydrate chains exerting an angiostatic function and impairing tumor growth
Source: Oncotarget. 2015 Dec 9;7(2):2022–37. doi: 10.18632/oncotarget.6515 (PMC4811514; doi:10.18632/oncotarget.6515)
Supplement: Supplementary file 1 [file oncotarget-07-2022-s001.pdf]

## SUPPLEMENTARY FIGURES

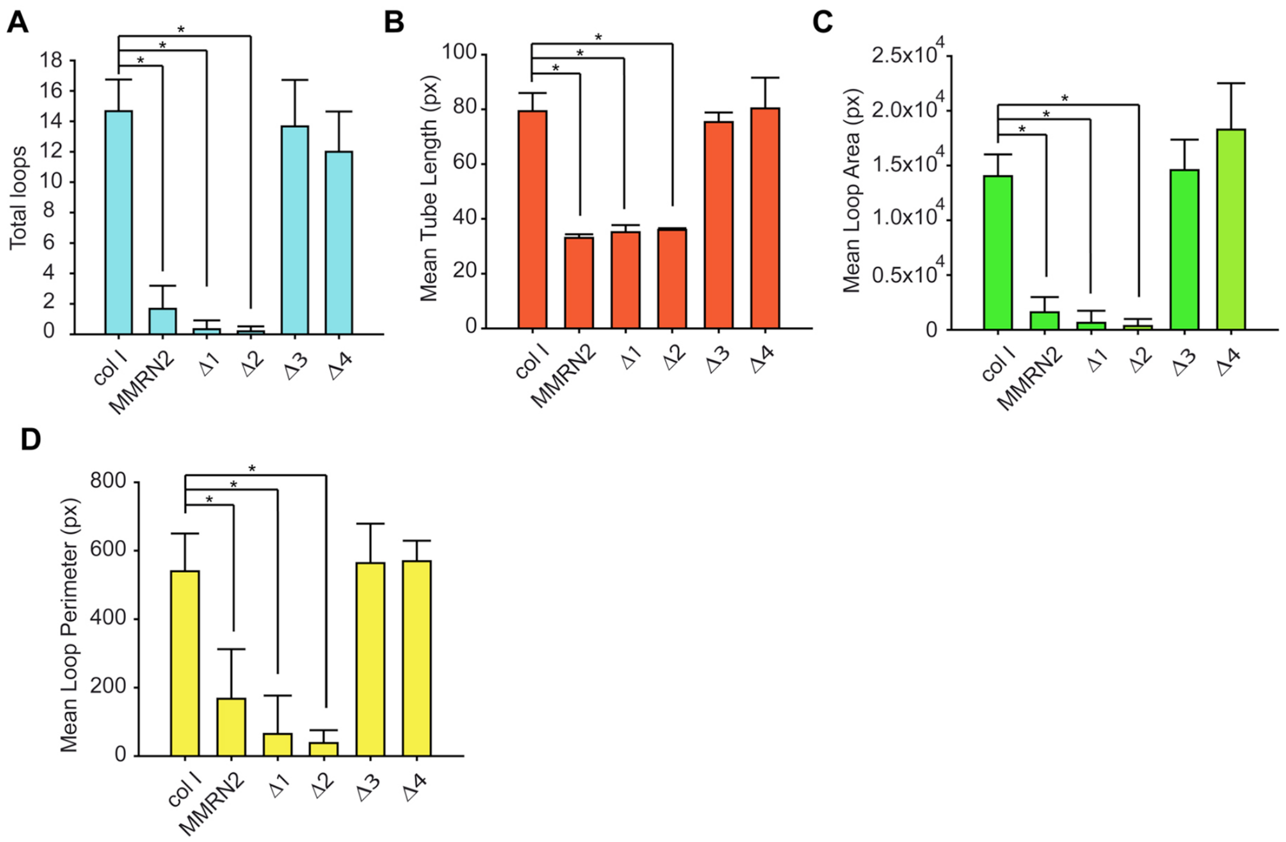


**Supplementary Figure S1: A, B, C and D. Graphs representing the analysis of respectively, the total loops (*P < 0.001), the mean tube length (*$P$ < 0.012), the mean loop area (*$P$ < 0.001) and the mean loop perimeter (*$P \leq$ 0.001) of the Matrigel tubulogenesis assay reported in Figure 2 as assessed with the Wimasis tube analysis software.** $P$ values were obtained with the ANOVA one way analysis of variance and graphs represent the mean ± SD obtained from at least three experiments.

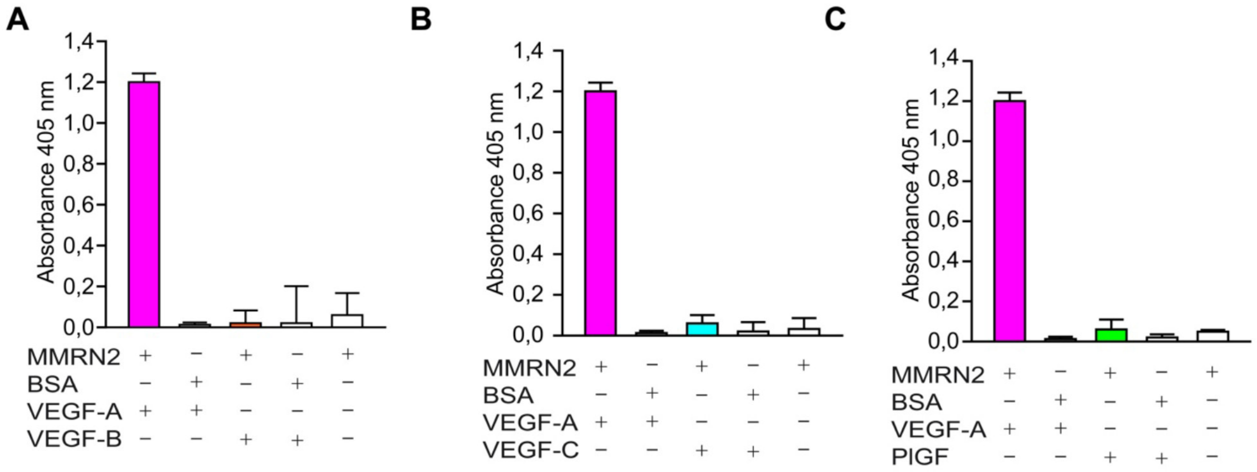


**Supplementary Figure S2: A, B, C. Graphs representing the solid phase analysis of the interaction of MMRN2 with the VEGF-A family members VEGF-B$_{167}$, VEGF-C and PlGF-1, respectively.** Unlike VEGF-A, no detectable binding was found for the other members of the family in this test. BSA was used as negative control.

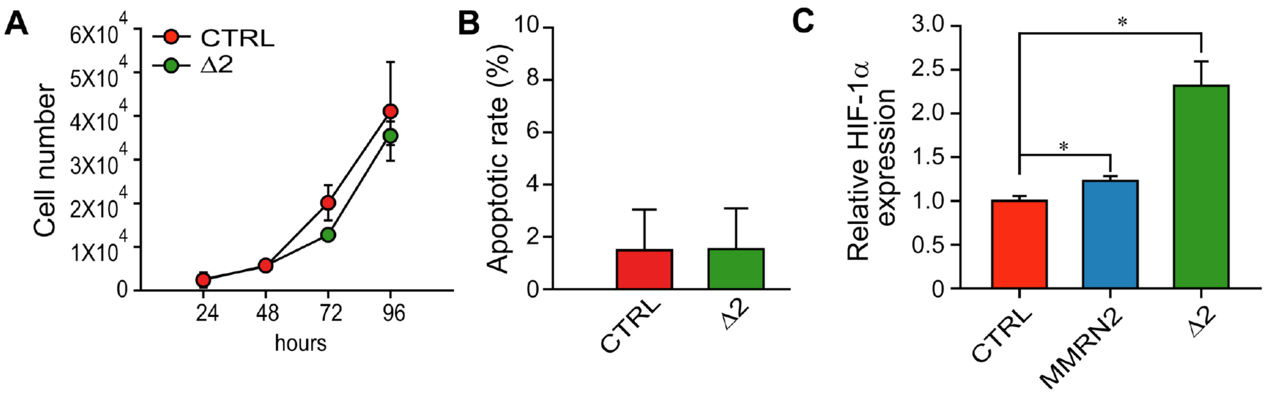


**Supplementary Figure S3: A. Graph reporting the growth curve of mock- or Δ2-transfected cells, as assessed by counting.** No significant changes in the growth of HT1080 cells ectopically expressing the Δ2 deletion mutant were detected. **B.** Graph reporting the similar % of apoptotic cells following transfection of HT1080 cells with the empty vector (CTRL) or ß2 deletion mutant construct, as assessed by TUNEL assay. **C.** Graph reporting the RealTime PCR analysis of HIF-1α expression in control (CTRL) or MMRN2 and Δ2 deletion over-expressing tumors (*$P < 0.001$). *P* values were obtained with the ANOVA one way analysis of variance and graphs represent the mean ± SD obtained from at least three experiments.

```
140       150       160       170       180       190
AIPEPADPGDSHQEPQDGPVSFKPGHLAAVINEVEVQQEQQEHLLGDLQNDVHRVADSLP      Δ2
CCCCCCCCCCCCCCCCCCCCCCCCCCCCHCCHHCHHHHHHHHCCCCCCCCCCCCCCCCCC      BPS
CCCCCCCCCCCCCCCCCCCCCCCCCCCCCCCCCCCCCCCCCCCCCCCCCHHHHHHHHHHH      K_S

    200       210       220       230       240       250
GLWKALPGNLTAAVMEANQTGHEFPDRSLEQVLLPHVDTFLQVHFSPIWRSFNQSLHSLT       2
CCCCCCCCCCCHHHHHHHHCCCCCCCCCCCCCCCCEECCEECECCEEEECCCHHCHHHHC      BPS
HHHHHCCCCCCCCCCCCCCCCCCCCCCCCCCCCCCHHHHHHHHHHHHHHHHCCHHHCCCC      K_S

    260       270       280       290       300      310
QAIRNLSLDVEANRQAISRVQDSAVARADFQELGAKFEAKVQENTQRVGQLRQDVEDRLH       2
HHHHHHHHHHHHHHHHHCCCCCCCCCCCCCCCCCCCCCHHHHHHECCCCCCCCCCCCCCC      BPS
HHHCCCCCCCCCCCCCCCCCCCCCCHHCCHHHHHHHHHHHCCCCCCHHHHHHCHHHHHHH      K_S

   320       330
AQHFTLHRSISELQADVDTK                                              2
CCHHCCCCCCCCCCHHHHHH                                              BPS
HHHHHHHCCCCCCCCCHHHH                                              K_S
```

**Supplementary Figure S4: Amino acid sequence of the Δ2 deletion mutant; the predicted regions of the deletion mutant presenting coiled-coil (C), alpha-helix (H) and beta-strand (E) protein structure are indicated as well as the Δ2-a (_), Δ2-b (_) and Δ2-c (_) additional fragments that were not synthesizable in 293-EBNA cells.** The predicted structure was obtained through the Burgess, Ponnuswamy and Sheraga (BPS) and the King and Sternberg (K_S) algorithms using the Biology Workbench sequence analysis tool (http://workbench.sdsc.edu/).
